# Supplementary figures and images for: Evaluation of the Genetic Diversity, Population Structure and Selection Signatures of Three Native Chinese Pig Populations
Source: Animals (Basel). 2023 Jun 16;13(12):2010. doi: 10.3390/ani13122010 (PMC10295185; doi:10.3390/ani13122010)

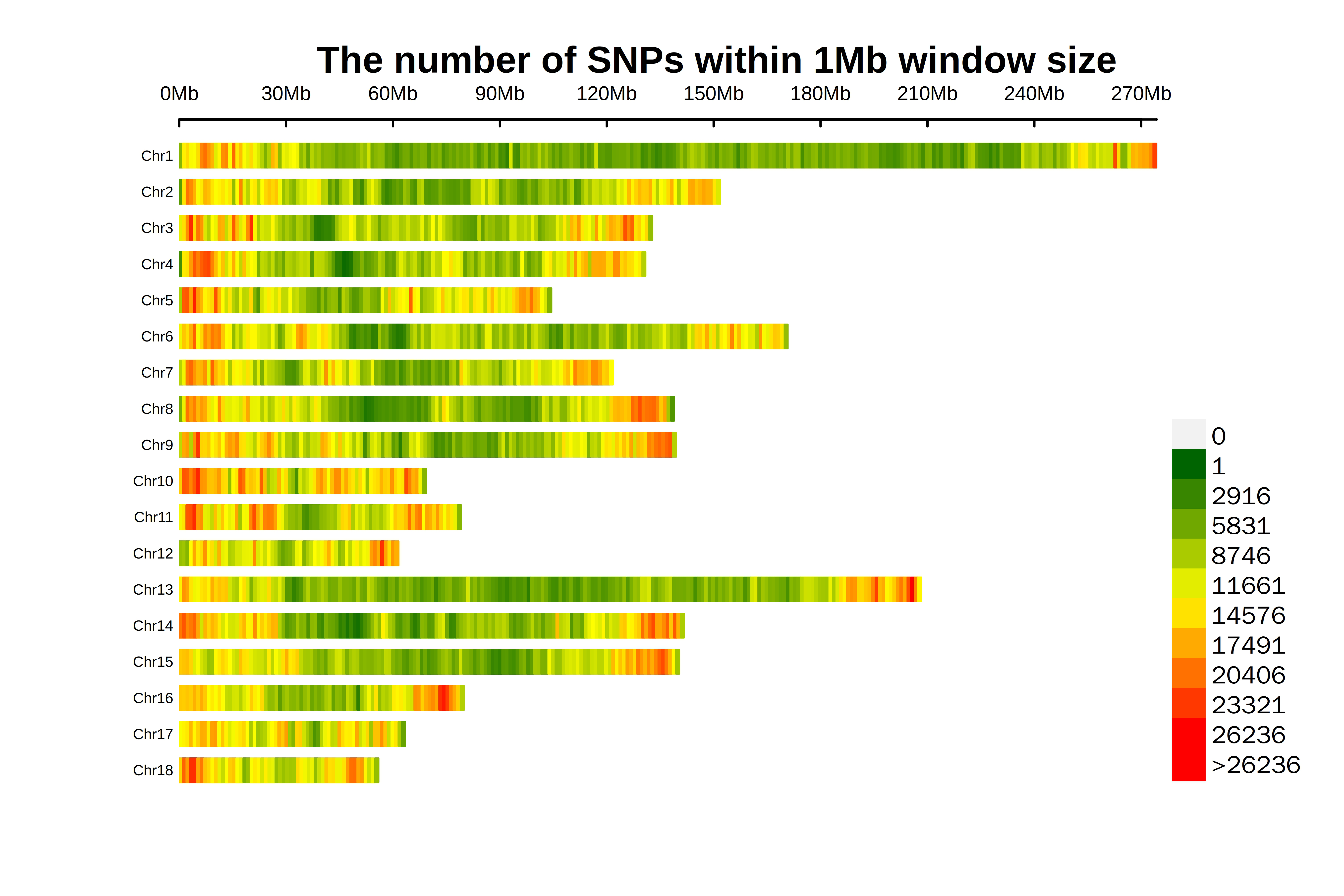

Supplement: Supplementary file 1 [file animals-13-02010-s001.zip › Figure S1.tif]
